# Prognostic Factors for Surgical Failure in Malignant Bowel Obstruction and Peritoneal Carcinomatosis

**DOI:** 10.3389/fsurg.2021.769658

**Published:** 2021-11-26

**Authors:** Claudio Lodoli, Marcello Covino, Miriam Attalla El Halabieh, Francesco Santullo, Andrea Di Giorgio, Carlo Abatini, Stefano Rotolo, Elena Rodolfino, Francesco Giovinazzo, Anna Fagotti, Giovanni Scambia, Francesco Franceschi, Fabio Pacelli

**Affiliations:** ^1^Surgical Unit of Peritoneum and Retroperitoneum Surgery, Fondazione Policlinico Universitario A. Gemelli, IRCCS, Rome, Italy; ^2^Department of Emergency Medicine, Fondazione Policlinico Universitario A. Gemelli, IRCCS, Rome, Italy; ^3^Università Cattolica del Sacro Cuore, Rome, Italy; ^4^Department of Radiology, Fondazione Policlinico Universitario A. Gemelli, IRCCS, Rome, Italy; ^5^General Surgery and Liver Transplantation, Fondazione Policlinico Universitario A. Gemelli, IRCCS, Rome, Italy; ^6^Division of Gynecologic Oncology, Department of Women and Children's Health, Fondazione Policlinico Universitario A. Gemelli, IRCCS, Rome, Italy

**Keywords:** malignant bowel obstruction (MBO), peritoneal carcinomatosis (PC), surgical palliation, ileostomy, palliative outcomes

## Abstract

**Introduction:** Patients with peritoneal metastasis frequently develop malignant bowel obstruction (MBO). Medical palliative management is preferred but often fails. Conversely, the role of palliative surgery remains unclear and debated. This study aims to identify patients who could benefit from invasive surgical interventions and factors associated with successful surgical palliation.

**Materials and Methods:** In this retrospective study, 98 consecutive patients who underwent palliative surgery for MBO over 5 years were reviewed. We evaluate as the primary outcome surgical failure to select patients who could benefit from palliative surgery, avoiding unnecessary surgery. A prognostic score was developed based on a logistic regression model to identify patients at risk of surgical failure. The score was evaluated for overall accuracy by receiver operating characteristic curve analysis.

**Results:** Palliative surgery was achieved in 76 (77.5%) patients. The variables that were found to be significant factors for surgical failure are recurrent disease (*P* = 0.015), absence of bowel obstruction (*P* < 0.001), absence of bowel distension (*P* < 0.001), and mesenteric involvement (*P* = 0.001) and retraction (P < 0.001). The absence of bowel distension (*P* = 0.046) and bowel obstruction (*P* = 0.012) emerged as independent predictors of surgical failure. Carcinomatosis level assessment for peritoneum score, based on these factors, was built to evaluate the risk of surgical failure.

**Conclusion:** Our proposed scoring system might help select patients most likely to benefit from palliative surgery.

## Introduction

Malignant bowel obstruction (MBO) secondary to peritoneal metastasis (PM) is a common consequence in patients with end-stage abdominal malignancies. The most frequent cancer etiologies of MBO are those of gastrointestinal and gynecological origin (1). The metastatic spread of cancer throughout the peritoneal surface often leads to bowel obstruction due to compression of the bowel by a tumor mass compression or infiltration into the bowel wall and mesentery. It can occur at single or multiple sites involving both small and large bowel.

Malignant bowel obstruction secondary to PM is a preterminal event associated with a poor prognosis (2, 3). When occlusive symptoms occur, this persuades to abstain from aggressive surgical treatment with curative intent, leaving room for palliation. Palliative conservative management is preferred (e.g., nasogastric tube, corticosteroid therapy, pain control, antiemetics, and antisecretory medications). However, when medical palliative management fails, the only remaining option is palliative surgery, (4, 5) usually consisting of ostomy creation.

Surgery may provide relief in distressed patients with intolerable symptoms improving their quality of life in terms of returning home and restoring oral intake, and in some cases allowing them to restart systemic chemotherapy. On the other hand, performing palliative surgery on poor surgical candidates like those with MBO is challenging because of the reduced life expectancy and the poor general conditions (6). This implies that even a standard surgical procedure can be associated with high postoperative morbidity and mortality (3, 7).

There is a lack of high-quality data on outcomes after surgery for MBO secondary to PM, and criteria for selecting patients who could benefit from palliative surgery remains spare. Furthermore, the heterogeneity of patients with MBO and the different treatment strategies do not allow to establish defined guidelines, appropriate patient selection, and tailored treatment (8, 9).

To guide surgeons and clinicians in their decision-making process, it is essential to obtain well-defined indications to avoid unnecessary and ineffective surgery. This work aims to identify criteria to select patients who could benefit from palliative surgery.

## Patients and Methods

The present work reports an observational, crosssectional study conducted in a large tertiary referral center for MBO. The study was retrospectively performed over 5 years between January 2014 and December 2018.

We reviewed the clinical records of all consecutive patients with MBO due to PM who underwent palliative surgery. The diagnosis of MBO was established on clinical and CT-scan findings (10–12).

We excluded patients with MBO for primary cancer without PM and patients who underwent conservative management and went back to the oncologists for palliative chemotherapy. Patient demographics, clinical parameters, tumor characteristics, treatment-related variables, and outcomes were collected.

All patients underwent medical palliative management, including fasting and nasogastric tube insertion, antiemetics, and antisecretory medications before surgery. The indication for surgery was the lack of response to medical treatment.

Clinical variables included for analysis were previous abdominal surgery, tumor histology, PM synchronous with the primary tumor, or peritoneal tumor recurrence.

All patients underwent a CT-scan evaluation to identify the site of bowel occlusion. A dedicated abdominal radiologist reviewed all CT scans of the abdomen. Radiographic findings included for analysis were the following:

- Bowel distension was defined as the presence of dilated bowel loops (small bowel caliber >2.5 cm; colon caliber >6 cm) proximally with the standard caliber or collapsed loops distally; bowel distension was categorized as: jejunal or upper-level dilatation, proximal ileal dilation, distal ileal dilatation, and colic dilatation. In case of dilatation in multiple bowel segments, the lowest level along the gastrointestinal tract of the patient was considered.
- Bowel obstruction was defined as the transition point, which is the physical point of obstruction at which dilated bowel proximally gives way to non-dilated bowel distally. Bowel obstruction was categorized as a jejunal or upper-level obstruction, proximal ileal obstruction, distal ileal obstruction, or colic obstruction. In case of multiple obstructions, the highest level along the gastrointestinal tract was considered for the patient.
- Mesenteric thickening was defined by an increased density of mesenteric fat (misty mesentery), a linear thickening of mesenteric reflections, and the presence of soft tissue rounded or speculated shape nodules in the broad mesentery fan and along the serosal surfaces. Mesenteric involvement was categorized as absent, partial, or diffuse.
- A mesenteric retraction was defined as a diffuse mesenteric thickening resulting in small bowel kinking and angulation.
- Moderate to severe ascites. Ascites were defined as moderate when there was fluid surrounding dependent regions and severe when fluid occupied the entire peritoneal cavity.

Operative approaches were tailored to each patient by the surgeon according to the history, clinical presentation, and preoperative imaging of the patient. Surgical palliation was defined as any surgical treatment that relieved the bowel obstruction (ostomy, intestinal bypass, or intestinal resection).

To select patients who could benefit from palliative surgery avoiding unnecessary and ineffective surgery, we decided to evaluate as a primary outcome the surgical failure, consisting of any ineffective laparotomy (“open and shut” laparotomy), as it was not possible to perform any surgical treatment to restore bowel intestinal function. After surgery, we assessed the ability to resume oral diet at the time of discharge from the hospital and the need for parenteral nutrition (NPT) in cases of surgical failure and in those cases where surgery has resulted in a short bowel. Postoperative complications were scored using the Clavien–Dindo classification (13). These were further grouped into minor (grade I and II) and major (grade III and IV) to perform statistical analysis.

## Statistical Analysis

Continuous variables are reported as median [interquartile range] and are compared at univariate analysis by Mann–Whitney *U*-test. Categorical variables are reported as absolute numbers (percentage) and are compared by the Chi-square test (with Fisher's test if appropriate).

Significant variables at univariate analysis were entered into a multivariate logistic regression model to identify independent risk factors for surgical failure. Association of factors to the risk of surgical failure is expressed as odds ratio (OR) [95% confidence interval].

### Score Building and Evaluation

All the independent predictors of surgical failure were analyzed to build a predictive scoring system (Carcinomatosis Level Assessment for Peritoneum (CLAP) score). Both variables included in the score were assigned a similar weight in the score.

The obtained CLAP score was evaluated for overall accuracy in identifying patients at risk of surgical failure by receiver operating characteristic (ROC) curve analysis. The sensitivity and specificity were identified for each score level by ROC analysis. The optimal dividing cut-off associated with CLAP score was obtained by Youden's index, and a two-sided *P*-value ≤ 0.05 was regarded as significant. Data were analyzed by SPSS v25® (IBM, Armonk, NY, USA).

## Results

### Patients Demographics

From January 2014 to December 2018, 127 patients diagnosed with bowel occlusion secondary to PM were admitted to Emergency Department (ED) and underwent palliative surgery in an emergency setting. CT-scan images of 29 patients were not available for review; thus, the study cohort consisted of 98 patients. Baseline characteristics are described in Table 1.

**Table 1:** Population demographics and baseline characteristics.

**Variable**	**All population**
	***n* = 98**
Age	56 [48–85]
Sex (Male)	22 (19.6%)
*Emergency department presentation*	
Abdominal pain	70 (71.4%)
Vomit	64 (65.3%)
Constipation	45 (45.9%)
Fever	13 (13.3%)
Diarrhea	5 (5.1%)
*Clinical conditions*	
Previous surgery	89 (90.8%)
Histology	
Gynecological	67 (68.4%)
Gastrointestinal	19 (19.4%)
Other	12 (12.2%)
Tumor recurrence	83 (84.7%)
*Radiological findings*	
*Level of bowel obstruction*	
No bowel obstruction	12 (12.2%)
Jejunal	17 (17.3%)
Proximal ileum	24 (24.5%)
Distal ileum	28 (28.6%)
Colic	17 (17.4%)
*Level of bowel distension*	
No distension	10 (10.2%)
Jejunal	19 (19.4%)
Proximal ileum	24 (24.5%)
Distal ileum	25 (25.5%)
Colic	20 (20.4%)
*Mesenterial thickening*	
No thickening	10 (10.2%)
Partial thickening	47 (48.0%)
Diffuse thickening	41 (41.8%)
*Mesenterial retraction*	35 (35.7%)
*Moderate/severe ascites*	30 (30.6%)
Discharge to hospice	48 (49.0%)
Re-alimentation	67 (68.4%)
NPT^§^	60 (61.2%)
In hospital death	3 (3.1%)
Length of hospital stay	16.5 [10.4–25.8]

§ *Need of parenteral nutrition*.

### Radiological Assessment

Bowel distension was present in almost all patients. In detail 19 (19.4%) patients had jejunal distension, 24 (24.5%) patients had proximal ileal distension, 25 (25.5%) patients had distal ileal distension, and 20 (20.4%) patients had colic distension.

In 12 (12.2%) patients, a point of bowel obstruction was not identified. Seventeen (17.3%) patients had jejunal obstruction, 24 (24.5%) patients had proximal ileal obstruction, 28 (28.6%) patients had distal ileal obstruction, and 17 (17.3%) patients had colic obstruction. There was a partial mesenteric involvement in 47 (48.0%) patients and diffuse mesenteric involvement in 41 (41.8%) patients. There was mesenteric retraction in 35 (35.7%) patients (Table 1).

### Surgical Procedure

In all individuals, diffuse peritoneal carcinomatosis was documented intraoperatively. In 69 (70.4%) patients, palliative surgery consisted of stoma creation. Of the 69 patients with ostomies created, five (7.2%) were colostomy. Ileostomy was performed in 43 (62.3%) patients and jejunostomy in 21 (30.4%) patients. Seven (7.1%) patients had a bowel resection, and in six patients a stoma creation was also performed. Seven (7.1%) patients received an intestinal bypass, and in three patients both a bypass and a stoma were created. Other surgical procedures, such as lysis of adhesion, were performed only in one patient. One patient underwent palliative tumor debulking (Table 2).

**Table 2:** Palliative surgical procedures.

**Palliative surgery**	**N% 76 (77.5%)**
Ostomy	69 (90.7%)
Jejunostomy	21 (30.4%)
Ileostomy	43 (62.3%)
Colostomy	5 (7.2%)
Bowel resection	7 (7.1%) 6 patients had also stoma creation
Intestinal bypass	7 (7.1%) 3 patients had also stoma creation
Palliative tumor debulking	1 (1.3%)
Other surgical procedures (lysis of adhesion)	1 (1.3%)

Ineffective (“open and shut” laparotomy) laparotomy occurred in 22 (22.4%) patients. These were considered surgical failure groups.

### Postoperative Outcomes and Scoring System

The median hospital length of stay after surgery was 16.5 [10.4–25.8]. There was no intraoperative death, but overall, three (3.1%) patients had in-hospital death. Palliative surgery was achieved in 76 (77.5%) patients. Sixty-seven (68.4%) patients were discharged tolerating oral diet. Sixty (61.2%) patients needed NPT regardless of the resumption of an oral diet. Forty-eight (49.0%) patients were discharged directly to hospice for terminal care, 50 (51.0%) patients were discharged home. Twenty-one (21.4%) patients developed postoperative complications, and among these nine (9.2%) were major complications (Table 3).

**Table 3:** Postoperative complications.

**Variable**	***N* (%)**
Complications	
*Yes*	21 (21.4)
*No*	77 (78.5)
Complication grade (Clavien-Dindo)	
*Grade I-II*	15 (15.3)
*Grade III-IV*	6 (6.1)
Pulmonary complications	4 (4)
Postoperative bleeding	1 (1)
Abdominal collection	3 (3)
Genitourinary infection	3 (3)
Surgical site infection	5 (5.1)
Intestinal perforation	2 (2)
Wound dehiscence	2 (2)
Stoma retraction	1 (1)

The following variables were tested by logistic regression analysis as a potential predictor of surgical failure: previous surgery, histology, tumor recurrence, type of palliative surgery, and radiological features (Table 4). On univariate analysis, the variables that were found to be significant factors for surgical failure are recurrent disease (*P* = 0.015), no bowel obstruction (*P* < 0.001), absence of bowel distension (*P* < 0.001), and mesenteric involvement (*P* = 0.001) and retraction (*P* < 0.001).

**Table 4:** Univariate comparison regarding surgery failure.

**Variable**	**Successful surgery** ***n* = 76**	**Surgery** **failure** ***n* = 22**	***p***
Age^§^	55 [48–65]	58 [51–66]	0.439
Sex (Male)	18 (20.5%)	4 (16.7%)	0.679
*Emergency department presentation*			
Abdominal pain	55 (72.4%)	15 (68.2%)	0.702
Vomit	53 (69.7%)	11 (50.0%)	0.087
Constipation	32 (42.1%)	13 (59.1%)	0.159
Fever	8 (10.5%)	5 (22.7%)	0.137
Diarrhea	4 (5.3%)	1 (4.5%)	1.000
*Clinical conditions*			
Previous surgery	69 (90.8%)	20 (90.9%)	0.986
Histology			
Gynecological	51 (67.1%)	16 (72.7%)	0.294
Gastrointestinal	17 (22.4%)	2 (9.1%)	
Other	8 (10.5%)	4 (18.2%)	
Tumor Recurrence	68 (89.5%)	15 (68.2%)	0.015
*Radiological findings*			
*Level of bowel obstruction*			
No bowel obstruction	1 (1.3%)	11 (50%)	
Jejunal	11 (14.5%)	6 (27.3%)	
Proximal ileum	20 (26.3%)	4 (18.2%)	<0.001
Distal ileum	27 (35.5%)	1 (4.5%)	
Colic	17 (22.4%)	0	
*Level of bowel distension*			
No distension	1 (1.3%)	9 (40.9%)	
Jejunal	13 (17.2%)	6 (27.3%)	
Proximal ileum	20 (26.3%)	4 (18.2%)	<0.001
Distal ileum	22 (28.9%)	3 (13.6%)	
Colic	20 (26.3%)	0	
*Mesenterial thickening*			
No thickening	7 (9.2%)	3 (13.6%)	
Partial thickening	44 (57.9%)	3 (13.6%)	0.001
Diffuse thickening	25 (32.9%)	16 (72.7%)	
*Mesenterial retraction*	20 (26.3%)	15 (68.2%)	<0.001
*Moderate/severe ascites*	21 (27.6%)	9 (40.9%)	0.234
*Outcomes*			
Discharge to hospice	28 (36.8%)	20 (90.9%)	<0.001
Re-alimentation	66 (86.6%)	1 (4.5%)	<0.001
NPT^§^	39 (51.3%)	21 (95.5%)	<0.001
In hospital death	2 (2.6%)	1 (4.5%)	0.538
Length of hospital stay	16.0 [10.1–26.0]	17.3 [12.0–24.0]	0.583

§ *Need of parenteral nutrition*.

In the multivariate model, only the absence of bowel distension (*P* = 0.046) and the absence of bowel obstruction (*P* = 0.012) emerged as independent predictors of surgical failure (Table 5).

**Table 5:** Multivariate analysis (logistic regression) of factor associated surgery failure.

**Variable**	**β**	**SE**	***P* value**	**Odds ratio (CI 95%)**
Bowel obstruction level	1.042	0.417	0.012	2.83 (1.25–6.41)
Bowel distension level	0.753	0.378	0.046	2.12 (1.01–4.45)
Mesenteric thickening	0.994	0.623	0.111	2.72 (0.80–9.16)
Recurrence (vs. primitive tumor)	−1.183	0.894	0.186	0.31 (0.05–1.77)
Mesenteric retraction	0.493	0.705	0.484	1.64 (0.41–6.52)

*Logistic regression model had an overall prediction of 77.6% (Model Chi2 = 43.74;−2log-likelihood = 60.636). Constant was included in the model*.

**Figure 1:**
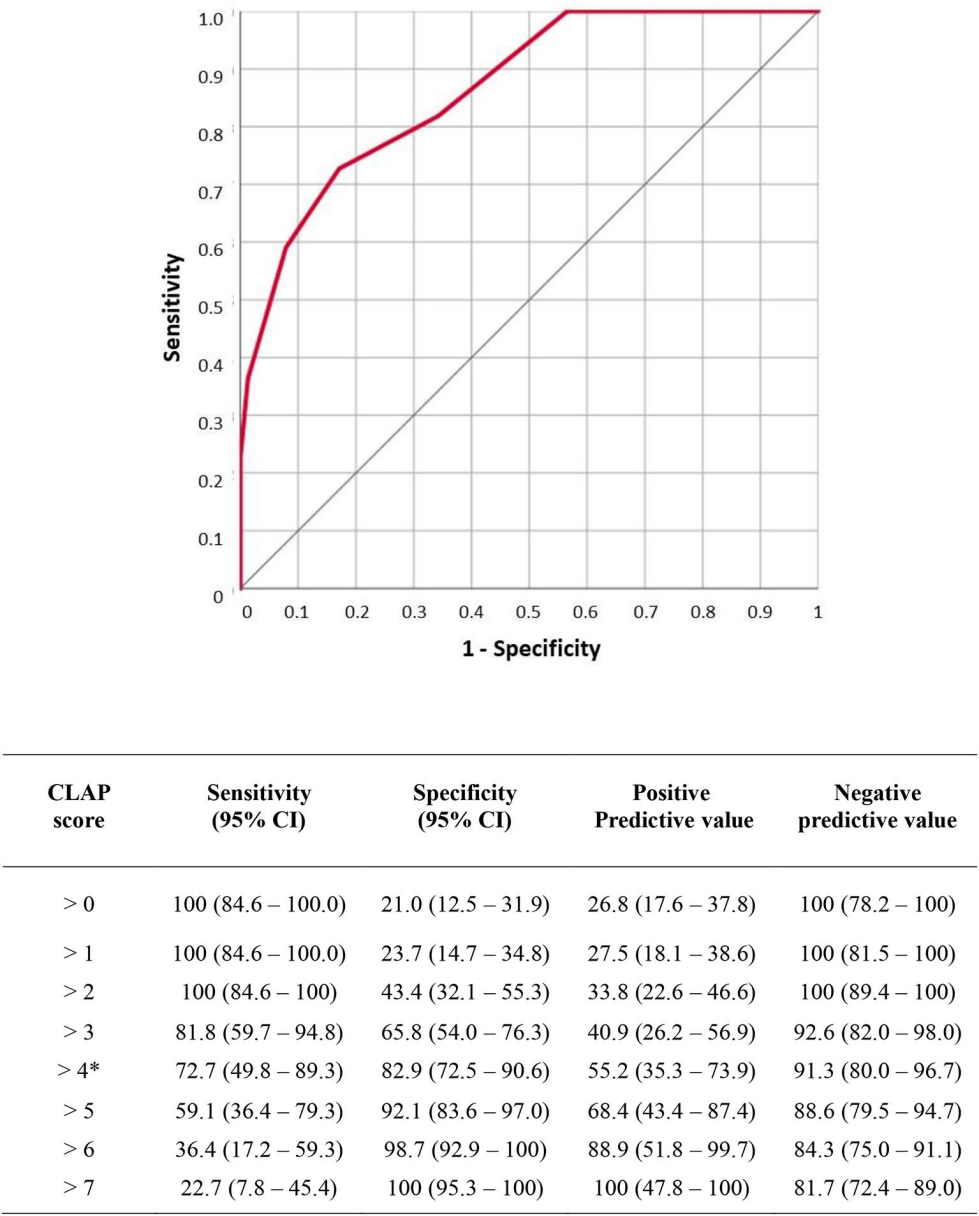
Receiver operating characteristic (ROC) analysis of carcinosis level assessment for peritoneum (CLAP) score. Area under ROC curve is 0.866 (0.782–0.926) *p* value < 0.001. According to Youden index J the best discriminating value was >4. Sensitivity, specificity, negative predictive value and positive predictive value for each score level are reported in the table.

To build the score, we assigned a score from 0 to 4 according to the site of obstruction or distension identified in these two variables, thus resulting in a score ranging from a minimum of 0 to a maximum of 8 points (Table 6). ROC analysis of the CLAP score is shown in Figure 1. Area under ROC curve is 0.866 (0.782–0.926) and *P*-value < 0.001. According to Youden index *J*, the best discriminating value in which the probability of surgical failure is high was >4.

**Table 6:** Point assigned to each condition to build the carcinosis level assessment for peritoneum (CLAP) score.

**Factor**	**Points assigned**
*Level of bowel distension*	
Colic	0
Distal ileum	1
Proximal ileum	2
Jejunal or more proximal	3
Non-evidenced	4
*Level of bowel obstruction*	
Colic	0
Distal ileum	1
Proximal ileum	2
Jejunal or more proximal	3
Non-evidenced	4

## Discussion

Bowel obstruction due to peritoneal carcinomatosis often shows up in patients with end-stage malignancies, who have already had several surgeries and have undergone multiple chemotherapy lines (14). Considering this, any medical specialist facing an MBO patient has to keep in mind the “first do no harm” Hippocratic oath and start with the least invasive treatment (15, 16). However, conservative management alone is not always the resolutive treatment, and when the bowel obstruction is complete, it is doomed to fail (17–19).

In this palliative context, surgery can be considered an option to relieve the unbearable symptoms of patients with MBO and, in some cases, as a bridge to restart chemotherapy treatment. Heyler et al. reported that surgery for MBO can facilitate palliative chemotherapy and, in a subset of patients it is associated with a significantly longer median survival of 10.3 months (20). However, surgery is known to be associated with a high incidence of morbidity and mortality, so it should always be considered cautiously in these patients (6, 21). Severe complications have been reported in several studies. De Boer et al. showed that more than half (58.1%) of the patients developed postoperative complications with in-hospital mortality of 8.8%. In a systematic review, Olson et al. confirmed that mortality is relatively high (6–32%), and serious complications are common (7–44%) (3). In our series, the incidence of postoperative complications (21.4%) is consistent with the literature, suggesting the feasibility of palliative surgery.

The peritoneal spread of tumors involves performing surgery on terminally ill and fragile patients and implies technical difficulties that can bring even experienced surgeons to failure in the operative room consisting in a so-called “open and shut” laparotomy. Hence, in the perspective of patient-tailored medicine, it becomes mandatory to identify patients for whom it is worth trying surgery to have a real palliative benefit (1, 22). In the current medical literature, established criteria to identify patients who could benefit from surgery are still lacking (23, 24). Some studies found several prognostic factors to be predictive of 30-day and 60- day overall survival, like the one of Perri et al. (8) that suggests ascites estimate below 2 L, younger age, primary ovarian tumor, and higher blood albumin correlated with more prolonged postoperative survival. Other studies suggested scoring systems to determine which patients would benefit from surgery (9), aiming at identifying prognostic factors predictive of survival; however, none of the current scores in literature assesses the risk of surgical failure.

In our series, we found two radiological variables independently associated with surgical failure: the site of bowel obstruction and the level of bowel dilatation. The site of bowel obstruction and dilatation are two intuitive prognostic factors of surgical failure in MBO. More proximal is the first physical point of gastrointestinal obstruction; more remarkable is the chance of an ineffective surgical procedure due to the shorter jejunal mesentery compared with the ileal mesentery.

Indeed, the effect of a mesenteric thickening and retraction is more evident in the shorter jejunal mesentery. In those circumstances, even for an experienced surgeon, it is technically impossible to obtain a bowel mobilization to create an ostomy.

In our series, although the mesenteric thickening and retraction had a significant association with surgical failure at univariate analysis, they were not confirmed as independent predictors of bowel dilatation and obstruction. A possible explanation of this finding could be that bowel obstruction and dilatation are more easily and accurately detectable preoperatively by a CT scan.

However, the new and unexpected concept that arose from our analysis is that the absence of bowel obstruction and dilatation in patients with MBO with PM is associated with surgery failure. Considering the patients in whom the surgery failed, 50% did not have a bowel obstruction (Table 4). Moreover, we found a surgical failure in 91.6% (11/12) of patients without obstruction. A possible explanation is that the amount of disease and the infiltration of the intestinal wall could reduce the motility of the bowel leading to occlusion also without obstruction. The direct consequence of this finding is that we found that 40.9% of surgery failures presented no evidence of bowel dilatation (Table 4). Bowel dilatation is associated with the ability of the bowel to expand because of the occlusion. Noteworthy are thus the cases of MBO in which dilation does not occur because the disease infiltrates the bowel walls, which can no longer dilate. This case means that the peritoneal spread of the tumor is very advanced and the possibility of successful palliative surgery, even just creating an ostomy, is very low.

The CT assessment of bowel dilatation and the bowel obstruction levels were used to build the CLAP score ranging from a minimum of 0 to a maximum of 8 points. The score was created, assigning 0 to 4 points according to the level of bowel obstruction and the level of bowel dilation (Table 6). The more distal the obstruction and dilatation levels, the lower the score assigned. According to Youden index *J*, the best discriminating value in which surgical failure probability is high was CLAP > 4 (Figure 1). In patients with CLAP > 4 in our cohort, 70.1% had a surgical failure.

We propose this score as an easy-to-use tool that utilizes only two variables. The variables considered are based on radiological findings and are objective and reproducible in similar clinical contexts. The CLAP score could reasonably allow the surgeon to understand better if it is worth taking the patient to the operating room or manage conservatively. Nevertheless, we suggest that this score must not ignore the physical examination and the case-by-case patient assessment, which remains a fundamental part of the surgeon's evaluation, frequently based on surgeon training and experience, as highlighted by a recent survey (25).

### Study Limitations

As for any retrospective study, some limitations are worth considering. First, since the study is focused on surgical failure, we did not collect data on survival or overall quality of life after discharge. Another minor limitation is the heterogeneity of the study population, which includes patients with cancers of various origins, although they all had MBO. Furthermore, all patients underwent surgery because they ran out of treatment options. Therefore, we cannot compare with non-operative management or comfort care in terms of palliative outcomes. Finally, as a tertiary referral center covering a large geographical area in the center and south of Italy, those patients were surgically treated and referred back to their oncology center. Therefore, it was complicated to track the overall survival and the cause of death for every single patient.

## Conclusion

Malignant bowel obstruction due to PM is a preterminal event and involves an extreme and challenging choice both for clinicians and patients and their families. Our work aims to guide surgeons and all health providers facing patients suffering from MBO in their daily practice. These select patients are likely to benefit from surgery and avoid unnecessary and possibly harmful treatments. In the absence of established guidelines, the proposed CLAP score, associated with a case-by-case evaluation and an accurate physical examination, could be an easy to use and reproducible tool in this setting.

Further prospective studies and external validation are still needed to confirm our findings.

## Data Availability Statement

The raw data supporting the conclusions of this article will be made available by the authors, without undue reservation.

## Ethics Statement

Ethical review and approval was not required for the study on human participants in accordance with the local legislation and institutional requirements. Written informed consent for participation was not required for this study in accordance with the national legislation and the institutional requirements.

## Author Contributions

FP, GS, and FG: manuscript review. FS and AF: manuscript editing. MA and MC: manuscript preparation. MC and FF: statistical analysis. MC and ER: data analysis and interpretation. AD, SR, and CA: quality control of data and algorithms. MA and CA: data acquisition. CL and MA: study design and study concepts. All authors contributed to the article and approved the submitted version.

## Conflict of Interest

The authors declare that the research was conducted in the absence of any commercial or financial relationships that could be construed as a potential conflict of interest.

## Publisher's Note

All claims expressed in this article are solely those of the authors and do not necessarily represent those of their affiliated organizations, or those of the publisher, the editors and the reviewers. Any product that may be evaluated in this article, or claim that may be made by its manufacturer, is not guaranteed or endorsed by the publisher.
